# Transactions of the Association of American Physicians

**Published:** 1891-09

**Authors:** 


TRANSACTIONS OF THE ASSOCIATION OF AMERICAN PHYSICIANS. Fifth Session, held at Washington, D. C., May 13, 14, and 15, 1890. Volume V. Cloth, 8vo, pp.—275. Edited by I. MINIS HAYS, M. D., Recorder. Philadelphia: Printed for the Association. 1890.

The fifth volume of the Transactions of this distinguished Association does not differ greatly from those that have preceded it in make-up or in interest. It records in a plain, straightforward, readable manner the transactions of the Association at its Washington meeting last year, and contains many papers of unusual interest. The paper of Dr. Lusk, on Antisepsis in Midwifery, and that by Dr. Shakespeare, on What Can be Done to Limit the Prevalence of Tuberculosis in Man, are especially to be commended for their able handling of important topics. The volume is edited by an experienced recorder, and is printed by Dornan, who has an especial aptitude for bringing out society transactions in proper shape.

